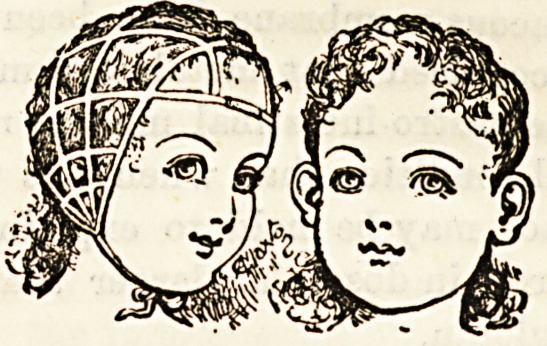# New Drugs and Appliances

**Published:** 1891-04-25

**Authors:** 


### NEW DRUGS, APPLIANCES, AND THINGS MEDICAL.

[All preparations, appliances, novelties, etc., of which a notice is desired, should be sent for The Editor, to care of The Manager, 140, Strand, London, W.C.]

### LISTERINE.

Messrs. Maw, Son, and Thompson have forwarded to us specimens of the above antiseptic fluid, which is prepared by the Lambert Pharmacal Company, St. Louis, U.S.A. It is stated to be a solution of "the essential antiseptic constituent of thyme, eucalyptus, baptisia, gaultheria, and Mentha arvensis, with a certain proportion of benzo-boracic acid." Its physical conditions are those of a nearly clear fluid of less s.g. than water, with a pleasant, refreshing odour, and warm aromatic taste. It is mixable in any proportion with water. In the pamphlet forwarded with the samples numerous experiments are detailed, proving its powers of antisepsis and prevention of germ multiplication with accompanying putridity. We have repeated some of these with the same results as those claimed. It quickly acts on bacterial growths, destroying them. Now, in what way can Listerine be used best? First, as it is not poisonous, it forms an admirable mouth wash and detergent for the teeth. When experimenting with it we had a case of ulcerative stomatitis with horribly fœtid breath. A lotion of one part to three of water frequently used quite removed the offensive odour. On the same day a case of dyspepsia with sour and stinking eructations presented itself; half drachm doses in hot water produced great relief at once and seem likely to cause a cure. A severe cut on the wrist was dressed with Listerine lotion and healed by first intention. We have also tried it as a deodorant after dressing a foul wound and found it entirely to remove the smell. These were common every day cases coming to our consulting room but the fluid answered well in each of them. Now it is quite impossible to compare Listerine with strong metallic disinfectants. These latter are poisonous, and so are incapable of internal use. In its limits we find Listerine a good and successful antiseptic, and can strongly recommend it. We fancy that it has a most useful post to fill in the dentist's consulting room, and certainly for domestic employment, where we can order it without the fear of accidents arising from the incautious use of corrosive fluids which after all, as generally used, are not more efficacious.

### NEW RAGOCYI EFFERVESCING SALINE FERRUGINOUS WATER.

We have received samples of the above water, with an analysis by Mr. R. H. Harland, F.I.C., F.C.S. The water is obtained from two wells on the New Ragocyi estate in Saxony. The analyses are as follows :—

| | No. 1. | No. 2. |
|---|---|---|
| Carbonate of lime...... | 19·25 | 15·05 |
| Sulphate of lime ...... | 11·90 | 7·47 |
| Sulphate of magnesia | 16·03 | 10·29 |
| Sulphate of potash ... | 4·48 | 4·90 |
| Sulphate of soda ...... | 21·98 | 15·75 |
| Chloride of sodium ... | 647·29 | 281·75 |
| Silica .................... | 2·10 | 1·82 |
| Carbonate of iron...... | 2·24 | 2·52 |
| Alumina .................. | ·07 | ·21 |
| Volatile matter ......... | 2·10 | ·35 |
| | 727·44 | 340·13 grs. per gallon. |

These waters have a great repute in Saxony for medical purposes. It will be noticed that the constituents are the same in each case, varying only in amount, No. 1 being nearly doubly as strong in saline ingredients, and so more efficacious as an aperient, whilst No. 2 possesses a rather larger proportion of iron. They are both remarkably highly charged with carbonic acid, so much so that on opening a bottle the water effervesces very powerfully. To the taste they are both pleasant and refreshing, No. 2 especially forming an agreeable table-water. No. 1 is a most excellent ferruginous laxative, and will be found very efficacious in cases of anæmia with constipation. Patients of this kind, mostly young girls, quickly tire of the routine dosing with the officinal preparations of iron, liquid or solid, with variations in the way of Blaud's pills, combined with saline purgatives. When they find that they can take their iron and salines in the form of a pleasant effervescing water, we fancy that there will be a considerable demand for the New Ragocyi. Again, cases of Bright's disease requiring chalybeate treatment will find in the No. 2 spring a table-water at once agreeable and possessing the required agent in its composition. We would give one hint for table-use: Do not mix a strongly astringent wine with the water, as the tannin of the wine will neutralise the good effect of the iron. We shall watch with considerable interest the results of treatment with this natural tonic, as we consider it one of the best ferruginous waters we have tested for some time.

### PETERMAN'S COCKROACH AND BEETLE FOOD (NON-POISONOUS).

Mr. J. F. Shorey, chemist, Farringdon Road, has sent us a specimen of this insect destroying food. Dr. Hassall's analysis shows its non-poisonous quality. We have submitted it to a practical test, and find that it does destroy cockroaches in large quantities, and consequently can confidently state that it is a success. This food, being non-poisonous and effective as a vermin killer, should commend itself to housekeepers all the world over.

### CLAXTON'S PATENT EAR CAP.

This is an excellent little invention, patented by Miss Adelaide Claxton, 62, Strand. It consists of a series of tapes fastened on to lengths of elastic webbing, and so arranged that the whole forms an open-meshed cap, tying under the chin. The meshes are closer over the ears. When in position a uniform elastic pressure is exerted over the head, and consequently over the ears. This pressure, if regularly maintained, will cause the growing cartilage of the ear to so mould itself that the ears are prevented from standing out in a disfiguring way. The cap also tends to keep the child's mouth shut, so preventing the habit of snoring. This, in the absence of naso-pharyngeal obstruction, will serve to obviate a very disagreeable habit which some children seem to acquire after being obliged to breathe for a time through the mouth because of nasal obstruction from a cold. We have tried the cap forwarded to us on a child, and find that it answers its purpose admirably. It is sightly, cool, easily adjusted, and effects its purpose of keeping the outstanding ears flat without being painful to the wearer. We consider the inventor has succeeded in producing an article which is effective, cheap, and so must fill a useful place in the nursery.

## Figures and Tables

**Figure f1:**